# Persistence of *Schistosoma haematobium* transmission among school children and its implication for the control of urogenital schistosomiasis in Lindi, Tanzania

**DOI:** 10.1371/journal.pone.0263929

**Published:** 2022-02-15

**Authors:** Vivian Mushi, Abdallah Zacharia, Magdalena Shao, Marycelina Mubi, Donath Tarimo

**Affiliations:** 1 Department of Parasitology and Medical Entomology, School of Public Health and Social Sciences, Muhimbili University of Health and Allied Sciences, Dar es Salaam, Tanzania; 2 Department of Environmental and Occupational Health, School of Public Health and Social Sciences, Muhimbili University of Health and Allied Sciences, Dar es Salaam, Tanzania; University of Ibadan, NIGERIA

## Abstract

**Background:**

Despite twelve rounds of school-based preventive chemotherapy for schistosomiasis in endemic areas of Tanzania such as Mtama district, Lindi: the burden of *Schistosoma haematobium* infection has remained highly conceivable due to re-infections. The factors associated with continuity of *S*.*haematobium* transmission in Mtama district, Lindi have not been fully established. This study investigated the burden and factors contributing to the ongoing transmission of *S*.*haematobium* infection in the endemic district of Mtama, Lindi.

**Methods:**

A quantitative cross-sectional survey was carried out among 649 school-age children in the Mtama district to determine the burden and factors associated with continuity of *S*.*haematobium* infection transmission. A single urine specimen was obtained from each pupil and tested for macro- and microhaematuria, presence of *S*.*haematobium* ova, as well intensity of infection; this was complemented with a survey of *Bulinus* spp snail intermediate hosts and their infectivity. A structured questionnaire was employed to gather information on individual and environmental risk factors for *S*.*haematobium* transmission. Summary statistics were computed for individual variables; while a univariate and multivariate logistic regression analysis was performed to assess the association between risk factors with *S*.*haematobium* infection.

**Results:**

Prevalence of *S*.*haematobium* infection by macro- and microhaematuria was 13.1% and 46.2% respectively. The prevalence of *S*.*haematobium* ova was 52.7%; intensity of infection was light in 53.1%, and heavy in 46.9%. Snail intermediate hosts were *Bulinus globosus* and *B*.*nasutus*, whose infectivity was 2.2% and 1.3%, respectively. Among the assessed risk factors, long residency (10–13 years) in the area was a significant risk factor for the continuity of *S*.*haematobium* transmission (AOR: 21.79, 95% CI: 1.37–346.4).

**Conclusions:**

The observed 52.7% prevalence of *S*.*haematobium* infection represents unacceptably high prevalence after 12 rounds of preventive chemotherapy. Therefore, an urgent need for the implementation of integrated multiple control interventions in the Mtama district; is considered to be imperative.

## Introduction

Urogenital schistosomiasis is an acute and chronic parasitic disease caused by blood flukes known as *Schistosoma haematobium (S*. *haematobium)*, which resides in the vasculature surrounding the urogenital system [1,2]. The disease is among the major public health problem in tropical and subtropical countries, especially where there is an inadequate supply of safe water (piped water), poor sanitation, and unhygienic practices such as open urination [3,4]. In endemic areas, the vulnerable population includes pre-school-aged children, school-aged children, and adults engaging in water contact occupations [2]. The infected children are at higher risk of poor cognitive function, poor growth, malnutrition, iron deficiency anemia, reduced school performance, and the development of hepatosplenic morbidities [5].

For transmission of urogenital schistosomiasis to occur, there must be contamination of the freshwater sources with viable *S*.*haematobium* eggs, presence of freshwater snails of the *Bulinus* species (spp), which serves as the intermediate host for the development of the cercariae, and human water contact, which expose them to the infective stage [6]. Globally, approximately 436 million people in 78 countries live in endemic areas that put them at risk of urogenital schistosomiasis, and over 112 million people are infected. Among the infected people, over 103 million suffer haematuria and dysuria, 100 million suffer minor bladder morbidity, 24 million suffer major bladder morbidity, 19 million suffer kidney problems, and 9.6 million have major hydronephrosis due to infection [2].

In Tanzania, urogenital schistosomiasis is widely distributed but more endemic along the eastern and south-eastern coasts, islands of Unguja and Pemba, and the hinterland areas of the north-western zones of the country [7,8]. Lindi being located along the southeastern part of the country has been a highly endemic area for urogenital schistosomiasis since 1987, with a prevalence of 58.9% among school-aged children [9]. Thus preventive chemotherapy with praziquantel was put in place in 2006 [10]. It is known that treatment with praziquantel does not prevent re-infection if exposure to the cercariae-infested water will continue [11]. Thus, despite twelve rounds of preventive chemotherapy with praziquantel the prevalence (23%) and intensity of schistosomiasis have remained high [12].

There is a paucity of information on the factors that have led to the persistently high prevalence of urogenital schistosomiasis among school-aged children in Lindi despite more than 12 rounds of praziquantel distribution and uptake. Therefore, this present study aimed to determine the current magnitude of the disease and the factors that might contribute to the persistent transmission of urogenital schistosomiasis among school-aged children in Mtama Lindi. The investigated factors were environmental factors (water, sanitation, and hygiene [WaSH] and malacological) and individual factors (knowledge, attitudes, and practices [KAP], and history of praziquantel uptake).

The findings will help neglected tropical diseases control managers in Lindi to make relevant programmatic changes needed to ensure sustainable control of urogenital schistosomiasis in Lindi. The findings will also provide practical information to policymakers that will aid in planning and implementing effective control strategies in Lindi.

## Materials and methods

### Study setting

The study area was Mtama District Council (DC), which is a new district council that was formerly known as Lindi DC. The district council is boarded to the north by Kilwa DC, to the south by the Mtwara Region, to the west by Nachingwea DC, and to the east by the Indian Ocean and Lindi Municipal Council. Mtama district council has an area of 5975 km^2^ with an approximate population of 194,143, whereby females are 102,496 and males are 91,647 [13].

The district experiences tropical climatic conditions characterized by an annual average rainfall of 910 mm and an average temperature of 26.3°C. The climatic condition of Mtama and the presence of both natural and man-made water sources favor the survival of the *Bulinus* snail (intermediate host of urogenital schistosomiasis).

**Fig 1:**
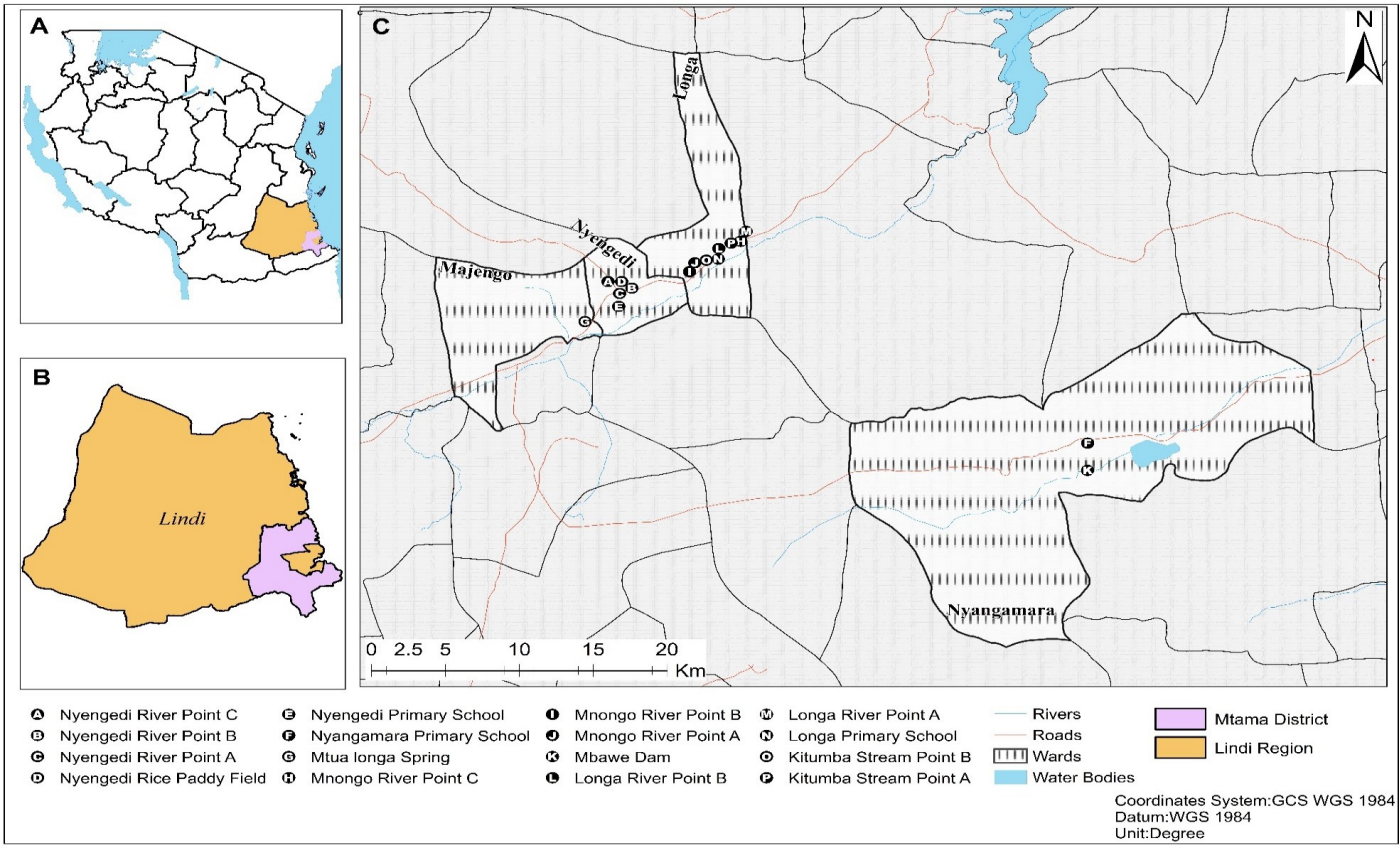
The map of Mtama district showing the schools and water bodies visited for parasitological and malacological survey. (The map was designed based on the data collected using Geographic Coordinate System (GPS) and mapped under GPS WGS 1984).

Mtama district council has 31 wards, and all wards are endemic to urogenital schistosomiasis. The economic activities include: agriculture, livestock rearing, and fishing. Some of these activities predispose the residents to the risk of acquiring urogenital schistosomiasis. Mtama district (Fig 1) was selected because of its history of urogenital schistosomiasis transmission since 1987, coupled with the scarcity of clean and safe water and higher proportions of households without an improved form of toilets [9,14,15].

### Study design

A school-based cross-section study involving a quantitative method of data collection was carried out in Mtama DC from April to May 2021 to investigate the current burden of urogenital schistosomiasis among school-age children, assess their KAP, and identify WaSH practices that predispose children to infection.

### Study population, inclusion and exclusion criteria

The study population was school children aged 6–17 years, attending school and permanent residents of the selected villages for at least two years. The sick children and those who used praziquantel three weeks before data collection were excluded from the study.

### Sample size and sampling procedure

**Fig 2:**
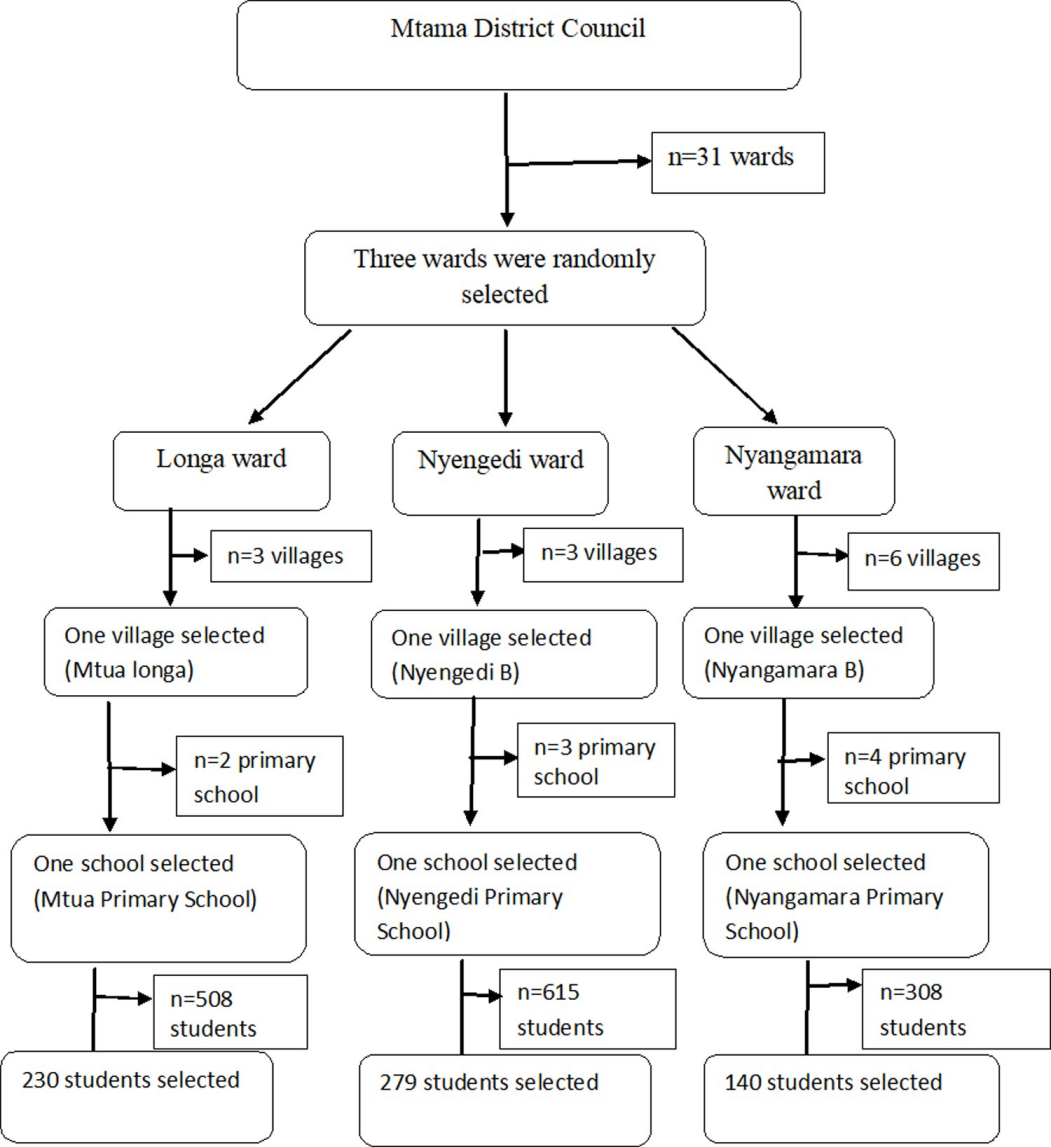
Flow chart of sampling technique.

The sample size of 649 school-aged children was estimated using the formula for cross-sectional surveys as described by Creswell [16]. This study used a multi-stage sampling technique to obtain a representative sample. Sampling was done in 3 stage cluster sampling technique as described in details in Fig 2. In each of the selected schools, the total number of students from standard one to seven was obtained, which helped to determine the number of students to be recruited from each school. Also, in each school, the total number of students per class was obtained from the head teacher’s offices so as to calculate the representative number of students to be selected from each class. The individual students to be recruited in each class were selected by random sampling using the lottery method.

### Parasitological survey

#### Urine samples collection and laboratory analysis

From each of the selected school aged children, a single urine sample was collected first followed by an interview. The children were given labeled containers with ID numbers for the collection of fresh urine samples. Samples were collected between 10:00 hours and 14:00 hours which is the optimum time for egg passage. The collected urine samples were examined immediately macroscopically by their color whether there is visible haematuria or none. Then urine reagent strips (dipstick) were used to detect microhaematuria. The dipsticks were dipped into containers that contain urine for 60 seconds to detect the presence of blood. In order to estimate the amount of blood in the urine, the color change of the dipstick was compared with the color chart on the bottle label. The results were recorded as positive or negative.

The presence of *S*.*haematobium* eggs in urine was examined using urine filtration technique. The procedures for each urine sample were as follows: the poly carbonate filter was placed in a filter holder, and then the urine sample was shaken and mixed before drawn 10 milliliters into a syringe. The filter holder was then attached to the end of a 10 milliliters syringe, and plunger of the syringe was pulled down to push all the urine through the filter to a beaker. The syringe was detached from the filter unit, then unscrewed and with the use of tweezers the filter was removed and placed inverted, onto the glass microscope slide. One drop of Lugol’s iodine was added to make eggs easily visible under the microscope. Then slide was examined under a microscope at a low power (x40). The eggs of *S*. *haematobium* were characterized with a terminal spine and counted manually [17]. Infection load was recorded as the number of eggs per 10ml of urine and categorized according to the World Health Organization (WHO) guideline of either light intensity 1–50 eggs/10mls) or heavy intensity (≥ 51 eggs/10ml of urine) [18].

### Malacological survey

A preliminary snail survey was done in the selected three villages rivers, dams, ponds, irrigation schemes, and streams where people do their domestic chores, collect water, swim, and play in the water (children). The areas were identified based on the information gathered during interviews with the children. A total of 15 sites were visited and sampled in the three villages in which sampled schools were located. The snails were collected between 09:30 am and 12:30 pm and the average time for snail collection per site was 20 minutes. The scooping and manual hand picking techniques were used to collect the snails. The scooping nets were dipped in water to collect the snails, then a scooper was used to pick the snail from the net, and placed them in labeled plastic containers containing water and aquatic plants from the sites for transferring the snail alive.

**Fig 3:**
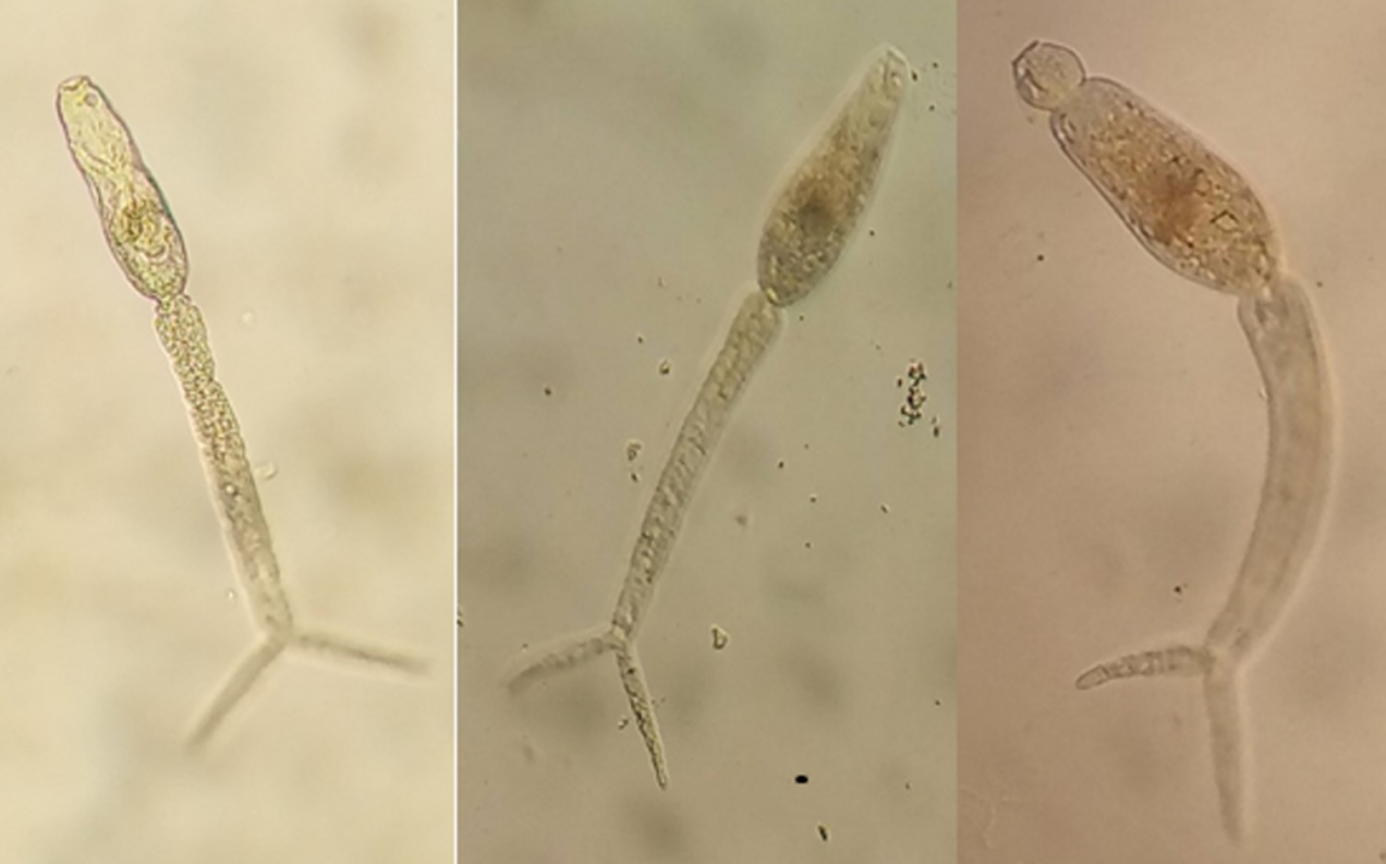
The bifurcate cercariae shed by the snails collected in Mtama.

Snails were identified to species level based on the morphological characteristics of their shells using standard identification keys [19,20]. The presence or absence of cercariae shedding was confirmed by using an emergent method of cercariae release. Each identified snail was placed in a separate beaker containing 10 to 20 milliliters of distilled water and exposed to sunlight for 2 hours to induce cercarial shedding. Then a microscopic examination of the samples to check for the emergent cercariae was done. The bifurcate cercariae were used to indicate the originality of the mammalian cercariae (Fig 3) [21].

### Questionnaire survey

A self-constructed pre-tested structured questionnaire was employed to collect information from school-aged children. The collected information included: socio-demographic data such as sex, age, grade and years of residency, water contact behaviors of the children, WaSH practices that could lead to transmission, participation in praziquantel treatment and knowledge, attitude and practices on urogenital schistosomiasis. The questions on the WaSH focused on water sources, availability, sanitation facilities, and personal hygienic practices. An English and Kiswahili version of the questionnaire is supplied as S1 and S2 Files.

### Data management and statistical analysis

Data collected was checked for completeness prior to coding, then entered, and analyzed using statistical package for the social sciences (SPSS) version 23 (IBM Corp., Armonk, NY, USA). Descriptive statistics was conducted to summarize the categorical variables in frequency tables with the proportions and their 95% confidence interval (CI). The prevalence of macro and micro haematuria, urogenital schistosomiasis and intensity was summarized based on socio-demographic characteristics of children while the prevalence of cercariae shedding snails was summarized based on the snail sampling sites and villages/schools.

The Pearson’s chi-square test and their p-value were used for comparisons of proportions. Univariate logistic regression was used to assess the association between the prevalence of urogenital schistosomiasis with socio-demographics, KAP and WaSH factors. All independent variables with a P-value < 0.25 were subjected to multivariate logistic regression analysis to obtain adjusted odds ratios, and the level of significance was set at 5%.

In measuring knowledge, a scale of seven multiple choice questions carrying a total of 11 correct responses was formulated. The corrected response was scored 1 while incorrect response was 0. In each individual the scores were added to make one scale ranging from 0 to 11 points. The mean knowledge scores was used to develop cut off points and classified as follows: 9–11 points indicated high level of knowledge, 5–8 points indicated moderate level of knowledge and 0–4 points indicated low level of knowledge.

Attitudes and practices were measured with a five point-Likert scale. For attitudes, there were 10 statements each was rated from 1 to 5 points. The statements were tested first for the reliability analysis and Cronbach’s alpha value of 0.686 was obtained. Then, a sum score was calculated and scale was formed ranging from 10 to 50 points. The mean attitude score was used to categorize attitudes as positive and negative. Positive attitudes ranged from 35 to 50 points, while negative attitudes ranged from 10 to 34 points. For practice, there were 10 statements each rated from 1 to 5 points. The reliability analysis for 10 statements resulted in Cronbach’s alpha value of 0.789. Accordingly, all 10 statements were added and a scale was formed, which ranged from 10 to 50. The mean practice score was used to categorize practices as appropriate and inappropriate. Appropriate practices ranged from 35 to 50 points, while inappropriate ones ranged from 10 to 34 points.

### Ethical statement

The ethical approval was obtained from the Muhimbili University of Health and Allied Sciences Ethical Review Board (MUHAS-REC-12-2020-457). Permission to conduct the study in Mtama DC was obtained from all administrative authorities of the Lindi region and Mtama district. At the beginning of the study, the parents/guardians were contacted by the headteachers and informed about the study and the verbal consent for their children to participate in the study. The written informed consent was provided and signed by headteachers on behalf of school children whose parents/guardians consented to participation in the study. This consent procedure was approved by the Muhimbili University of Health and Allied Sciences Ethical Committee.

## Results

### Socio-demographic characteristics of the study participants

This study recruited a total of 649 school-aged children from classes one to seven. Of the total participants, more than half (53.2%) were females. The mean age of the children was 10.85 (±2.63) years. Nearly half (48.1%) of the participants have resided in Mtama for 10 to 13 years (Table 1).

**Table 1:** Socio-demographic characteristics of the study participants (n = 649).

Variable	n (%)	95% CI
**Sex**		
Males	304 (46.8)	43–50.8
Females	345 (53.2)	49.2–57
**Age (years)**	**10.85±2.63**	
6–9	207(31.9)	28.4–35.9
10–13	329 (50.7)	46.5–54.2
14–17	113 (17.4)	14.5–20.5
**Class**		
Class one	99 (15.3)	12.8–18.3
Class two	111(17.1)	14.2–20.5
Class three	99 (15.3)	12.6–18.2
Class four	83 (12.8)	10.2–15.3
Class five	107 (16.5)	13.9–19.2
Class six	74 (11.4)	8.9–14
Class seven	76 (11.7)	9.2–14.5
**Residency (years)**	**10.58±2.88**	
≤ 5	13 (2)	1.1–3.2
6–9	216 (33.3)	29.7–37.2
10–13	312 (48.1)	44–51.9
14–17	108 (16.6)	13.7–19.9
**Schools**		
Longa Primary School	230 (35.4)	31.7–39.1
Nyengedi Primary School	279 (43)	39.2–46.6
Nyangamara Primary School	140 (21.6)	18.7–24.8

### Prevalence of macro and micro haematuria, urogenital schistosomiasis and intensity stratified according to socio-demographic characteristics of the study participants

The prevalence of macrohaematuria, microhaematuria, and urogenital schistosomiasis was 13.1%, 46.2%, and 52.7%, respectively. Males had a higher prevalence of macrohaematuria (16.4%), microhaematuria (50%), and urogenital schistosomiasis (57.2%). Also, the children aged 14 to 17 years had a higher prevalence of microhaematuria (52.2%) and urogenital schistosomiasis (56.6%) compared to the rest. There was a statistically significant difference in the prevalence of urogenital schistosomiasis between sex (p = 0.03), years of residency (p = 0.028), and the schools (p<0.000). The overall mean intensity was 90.23±241.67 EPG. A higher intensity was observed in males (103.26(±253.53)) and age group 10 to 13 years (105.51 (±256.77)) (Table 2). According to the WHO criteria for the intensity classification, more than half (53.1%) of the participants had a light infection, and 46.9% had a heavy infection.

**Table 2:** Prevalence of macro and micro haematuria, urogenital schistosomiasis and intensity stratified according to socio-demographic characteristics of the study participants (n = 649).

Socio-demographics	Total	Prevalence of macrohaematuria n (%)	p-value	Prevalence of microhaematuria n (%)	p-value	Prevalence of urogenital Schistosomiasis n (%)	p-value	Average ova counts + SD
**Sex**								
Males	304	50 (16.4)	0.018	152 (50)	0.070	174 (57.2)	0.030	103.26(±253.53)
Females	345	35 (10.1)		148 (42.9)		168 (48.7)		78.74 (±230.48)
**Age (years)**								
6–9	207	22 (10.6)	0.184	71 (34.3)	<0.000	97(46.9)	0.120	80.73 (±227.71)
10–13	329	51 (15.5)		170 (51.7)		181 (55)		101.16 (± 252.17)
14–17	113	12 (10.6)		59 (52.2)		64 (56.6)		75.78 (±235.772)
**Class**								
Class one	99	11 (11.1)	0.719	30 (30.3)	0.011	43 (43.4)	0.297	79.55 (±225.84)
Class two	111	14 (12.6)		49 (44.1)		57(51.4)		108.36 (±267.96)
Class three	99	18 (18.2)		48 (48.5)		52 (52.5)		88.64 (±203.46)
Class four	83	11 (13.3)		37 (44.6)		46 (55.4)		118.76 (±298.50)
Class five	107	13 (12.1)		60 (56.1)		66 (61.7)		73.90 (±183.70)
Class six	74	7 (9.5)		35 (47.3)		39 (52.7)		64.15 (±213.69)
Class seven	76	11 (14.5)		41 (53.9)		39 (51.3)		96.95 (±293.92)
**Residency(years)**								
≤ 5	13	2 (15.4)	0.116	7 (53.8)	<0.000	8 (61.5)	0.028	65.46 (±161.99)
6–9	216	21 (9.7)		73 (33.8)		96 (44.4)		75.98 (±223.03)
10–13	312	51 (16.3)		164 (52.6)		175 (56.1)		105.51 (±256.77)
14–17	108	11 (10.2)		56 (51.9)		63 (58.3)		77.56 (±240.53)
**Schools**								
Longa Primary School	230	24 (10.4)	<0.000	117 (50.9)	<0.000	140 (60.9)	<0.000	57.51 (±142.94)
Nyengedi Primary School	279	53 (19)		144 (51.6)		156 (55.9)		149.38 (±328.06)
Nyangamara Primary School	140	8 (5.7)		39 (27.9)		46 (32.9)		26.09 (±102.14)
**Overall**		85(13.1%)		300(46.2%)		342(52.7%)		90.23±241.67

### History of schistosomiasis sickness, treatment and self-reported uptake of praziquantel among the study participants

More than half (59.8%) of the participants reported ever suffered urogenital schistosomiasis. Of the reported individuals, about two-thirds (68.6%) only were investigated at the health facility before treatment. Nearly a quarter (20.5%) of the participants did not swallow the praziquantel in the last round of the distribution, with the leading reason being not yet enrolled in standard one, hence, not qualified to take the praziquantel (Table 3).

**Table 3:** History of schistosomiasis sickness, treatment and self-reported uptake of praziquantel among the study participants (n = 649).

Variable	n(%)	95% CI
**Ever suffered urogenital schistosomiasis**		
Yes	388 (59.8)	56.0–63.6
No	261 (40.2)	36.4–44.0
**When suffered**		
Currently I am suffering	42 (10.8)	7.9–14
A month ago	28 (7.2)	4.8–10
More than 3 months ago but less than six months	37 (9.5)	6.7–12.9
More than six months ago	70 (18)	14.3–22.1
A year ago	148 (38.1)	33.1–43.1
Do not remember	63 (16.2)	12.8–19.8
**Investigated in the health facility**		
Yes	266 (68.6)	64.0–73.3
No	122 (31.4)	26.7–36.0
**Treated**		
Yes	347 (89.4)	86.1–92.4
No	41 (10.6)	7.6–13.9
**Have you ever swallowed praziquantel**		
Yes	545 (84)	80.9–86.6
No	104(16)	13.4–19.1
**Did you swallow praziquantel in the last round**		
Yes	512 (78.9)	76–82
No	133 (20.5)	17.6–23.4
Do not remember	4 (0.6)	0.2–1.2
**Reason for not swallowing praziquantel**		
Not yet enrolled in class one	99 (74.4)	66.7–81.5
Did not attend the school on the MDA day	21 (15.8)	9.6–23.2
Sick	6 (4.5)	1.4–8.1
Parent did not allow	5 (3.8)	0.8–7.6
New comer (no school MDA in the previous school)	2 (1.5)	0.0–3.8

### Knowledge on the urogenital schistosomiasis among study participants

All respondents had heard about urogenital schistosomiasis, with the majority (74.1%) knowing transmission of urogenital schistosomiasis occurs by swimming and playing in infested water. More than two-thirds (69.5%) of the participants correctly reported that haematuria is the symptom of urogenital schistosomiasis. Regarding the treatment and prevention, the majority reported schistosomiasis as a curable (85.1%) and preventable (73.5%) disease. The use of anti-schistosomal medicine was the most mentioned (21.4%) way of urogenital schistosomiasis prevention compared to the rest of preventive measures (Table 4).

**Table 4:** Knowledge on the urogenital schistosomiasis among study participants (n = 649).

Variable	n (%)	95% CI
**Heard of urogenital schistosomiasis**		
Yes	649 (100)	
No		
**Source of the information**		
Home	299 (46.1)	42.1–49.9
Mass media	26 (4)	2.5–5.4
School	211 (32.5)	28.8–36.1
Dispensary	66 (10.2)	8–12.6
Friend	32 (4.9)	3.4–6.8
Home and School	5 (0.8)	0.2–1.5
Mass media and Friend	10 (1.5)	0.6–2.5
**Mode of urogenital schistosomiasis transmission**		
By drinking dirty water	65 (10)	7.6–12.5
By swimming and playing in infested water	481 (74.1)	70.4–77.5
By shaking hands	13 (2)	1.1–3.2
By eating contaminated food	8 (1.2)	0.5–2.2
By playing with dirty soil	1 (0.2)	0.0–0.5
Do not know	81 (12.5)	10–15.2
**Do snails transmit urogenital schistosomiasis**		
Yes	395 (60.9)	56.5–64.6
No	140 (21.6)	18.5–25
Do not know	114 (17.6)	14.9–20.5
**Symptoms of urogenital schistosomiasis**		
Coughing	6 (0.9)	0.3–1.7
Itching of genitalia	22 (3.4)	2.2–4.6
Headache	9 (1.4)	0.6–2.5
Fever	7 (1.1)	0.3–2
Blood in the urine	451 (69.5)	65.8–72.7
Blood in the urine and itching	2 (0.3)	0.0–0.8
Blood in the urine and fever	2 (0.3)	0.0–0.8
Blood in feces	10 (1.5)	0.8–2.6
Stomach ache	44 (6.8)	4.9–8.8
Stomach ache and itching	3 (0.5)	0.0–1.1
Diarrhea	8 (1.2)	0.5–2.2
Do not know	85 (13.1)	10.6–15.9
**Is schistosomiasis cured**		
Yes	552 (85.1)	82.3–87.7
No	40 (6.2)	4.5–8.0
Do not know	57 (8.8)	6.6–10.9
**Ways to treat urogenital schistosomiasis**		
By swallowing tablets	502 (90.9)	88.5–93.4
By injection	45 (8.2)	5.7–10.5
By traditional medicine	2 (0.4)	0.0–0.9
Do not know	3 (0.5)	0.0–1.3
**Is schistosomiasis preventable**		
Yes	477 (73.5)	70.1–76.7
No	94 (14.5)	11.7–17.2
Do not know	78 (12)	9.7–14.6
**Ways to prevent urogenital schistosomiasis**		
Treatment using anti-schistosomal medicine	102 (21.4)	17.5–25
By avoiding contact with unprotected water bodies	99 (20.8)	17.3–24.4
Use of pipe water	49 (10.3)	7.6–13.1
Use of latrines	84 (17.6)	14.3–20.8
By improving of personal hygiene	46 (9.6)	6.9–12.3
Treatment using anti-schistosomal medicine and use of latrines	8 (1.7)	0.6–2.8
Treatment using anti-schistosomal medicine and use of pipe water	7 (1.5)	0.6–2.8
Use of pipe water and use of toilets	4 (0.8)	0.2–1.8
By avoiding contact with unprotected water bodies and use of latrines	7 (1.5)	0.4–2.5
Treatment using anti-schistosomal medicine, avoiding contact with unprotected water and use of latrines	13 (2.7)	1.4–4.3
Treatment using anti-schistosomal medicine, use of pipe water and use of toilets	5 (1)	0.2–2
By avoiding contact with unprotected water bodies, use of piped water and use of latrines	11 (2.3)	1.1–3.8
All of the mentioned measures	27 (5.7)	3.9–7.7
Do not know	15 (3.1)	1.5–4.9

### Classification of the levels of knowledge among the study participants

A low level of knowledge on urogenital schistosomiasis was observed among females (37.7%), age group 6 to 9 years (50.2%), and class one students (59.6%). There was a statistically significant difference between the levels of knowledge and all socio-demographic characteristics (p < 0.000) (Table 5). Of the total participants, 202 (31.1%) had a low level of knowledge, 421 (64.9%) had a moderate level of knowledge, and 26 (4%) had a high level of knowledge regarding urogenital schistosomiasis.

**Table 5:** Classification of knowledge levels according to socio-demographic characteristics (n = 649).

Socio-demographics	Low level of knowledge	Moderate level of Knowledge	High level of knowledge	p-value
**Sex**				
Males	72 (23.7)	221 (72.7)	11 (3.6)	< 0.000
Females	130 (37.7)	200 (58)	15 (4.3)	
**Age (years)**				
6–9	104(50.2)	102 (49.3)	1 (0.5)	< 0.000
10–13	79(24)	238 (72.3)	12 (3.6)	
14–17	19 (16.8)	81 (71.7)	13 (11.5)	
**Class**				
Class one	59 (59.6)	40 (40.4)	0 (0)	< 0.000
Class two	47 (42.3)	63 (56.8)	1 (0.9)	
Class three	31 (31.3)	67 (67.7)	1 (1)	
Class four	23 (27.7)	59 (71.1)	1 (1.2)	
Class five	20 (18.7)	81 (75.7)	6 (5.6)	
Class six	13 (17.6)	57 (77)	4 (5.4)	
Class seven	9 (11.8)	54 (71.1)	13 (17.1)	
**Residency(years)**				
≤ 5	4 (30.8)	9 (69.2)	0 (0)	< 0.000
6–9	108 (50)	107 (49.5)	1 (0.5)	
10–13	73 (23.4)	227 (72.8)	12 (3.8)	
14–17	17 (15.7)	78 (72.2)	13 (12)	
**Schools**				
Longa Primary School	33 (14.3)	174 (75.7)	23 (10)	< 0.000
Nyengedi Primary School	110 (39.4)	166 (59.5)	3 (1.1)	
Nyangamara Primary School	59 (42.1)	81 (57.9)	0(0)	

### Attitudes and practices on urogenital schistosomiasis among the study participants

**Fig 4:**
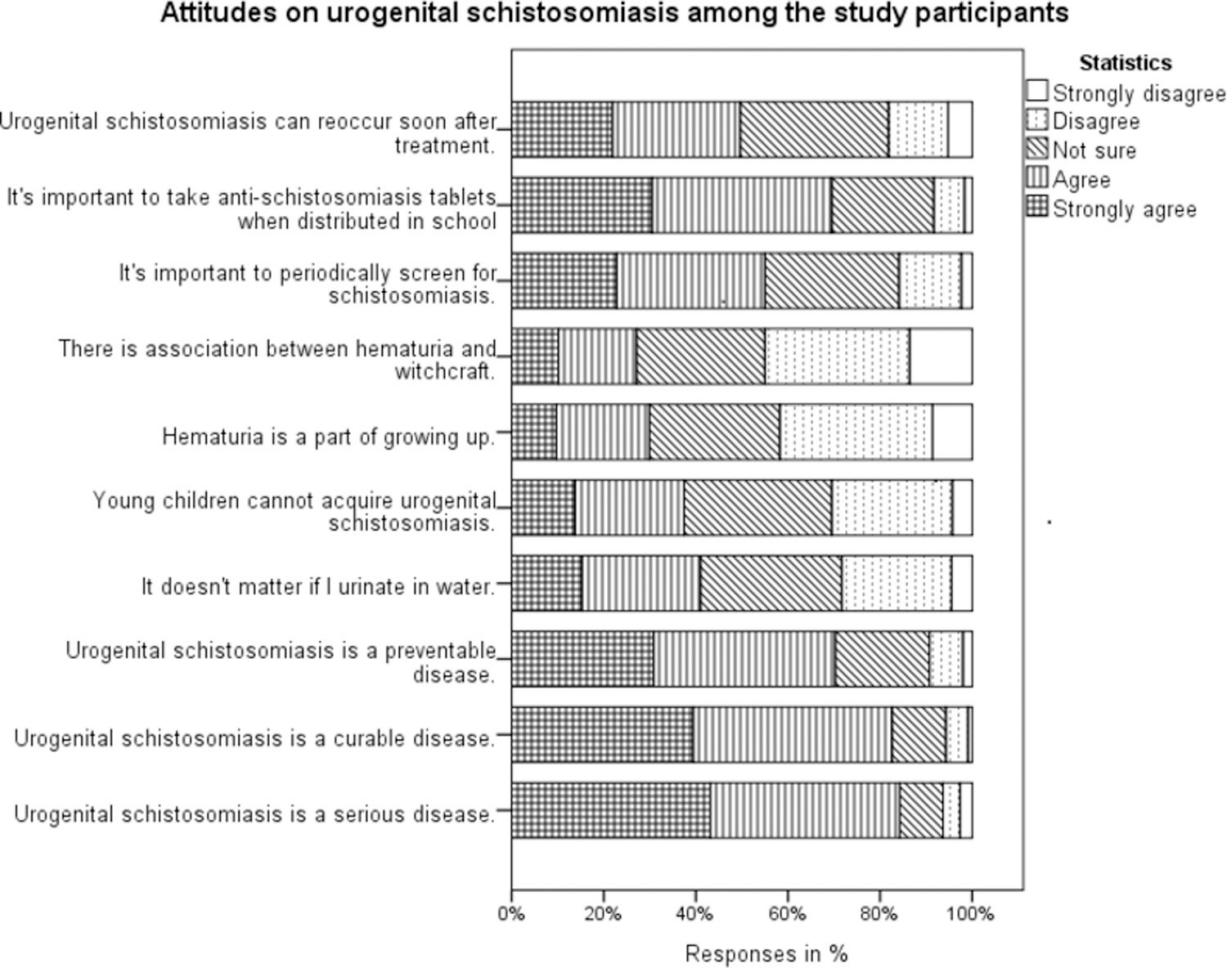
Attitudes on urogenital schistosomiasis among the study participants.

The majority (84.2%) of participants reported urogenital schistosomiasis as a serious disease. However, more than one-third (41.4%) of the participants believed practicing open urination was not a problem. Also, nearly one-third (32% and 32.2% respectively) of participants were not sure about the acquisition of the disease among under-fives and recurrence of urogenital schistosomiasis soon after treatment (Fig 4). Of the 649 participants, more than half (52.5%) had positive attitude, and the rest (47.5%) had negative attitude.

**Fig 5:**
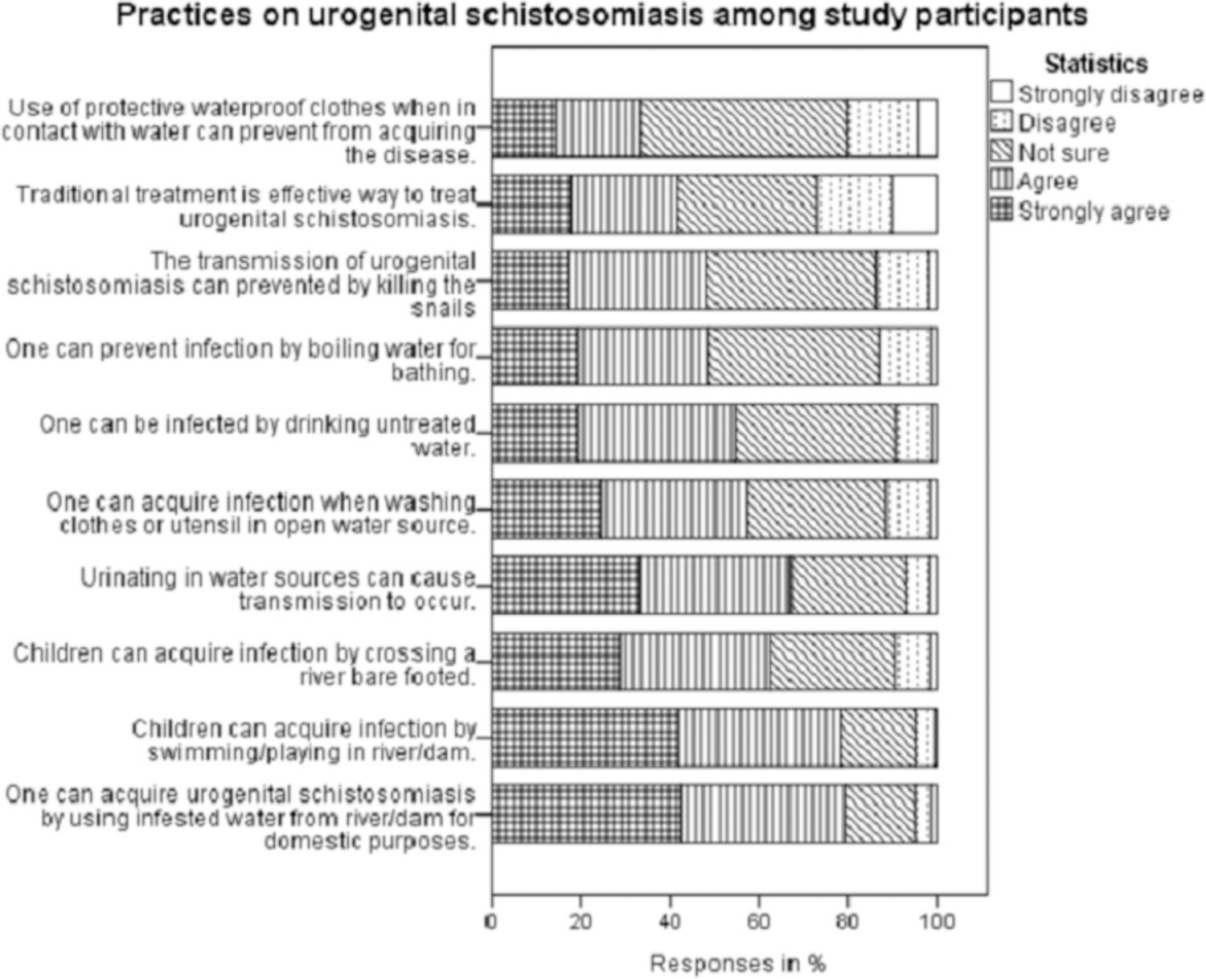
Practices on urogenital schistosomiasis among study participants.

The majority (78.3%) of participants reported that the practice of swimming or playing in a river/dam can cause one to be infected. More than one-third (37.9%) were not sure if the killing of snails will prevent urogenital schistosomiasis transmission. More than half (54.7%) of participants had a misconception that the practice of drinking untreated water can cause infection. Also, about 41% of the participants believed traditional treatment was an effective way to treat urogenital schistosomiasis compared to the modern treatment (Fig 5). Of the 649 participants, more than half (58.9%) had appropriate practices towards urogenital schistosomiasis, and the rest (41.1%) had inappropriate practices.

### Classification of attitudes and practices according to socio-demographic characteristics

The age group 6 to 9 years had a higher number of participants with negative attitudes (61.4%) and inappropriate practices (59.4%) towards urogenital schistosomiasis compared to the rest of the age groups. Also, class one students had a high number of participants with negative attitudes (71.7%) and inappropriate practices (59.4%) towards urogenital schistosomiasis compared to the rest of the classes. There was a statistically significant difference between the levels of attitudes and the age groups, classes, schools, and years of residency (p< 0.000), and practices with all social demographic characteristics (Table 6).

**Table 6:** Classification of attitudes and practices according to the social- demographic characteristics (n = 649).

Socio-demographics	Positive attitudes	Negative attitudes	p-value	Appropriate practices	Inappropriate practices	p-value
**Sex**						
Male	165(54.3)	139 (45.7)	0.406	195(64.1)	109 (35.9)	0.010
Female	176 (51)	169 (49)		187 (54.2)	158 (45.8)	
**Age (years)**						
6–9	80 (38.6)	127 (61.4)	< 0.000	84 (40.6)	123 (59.4)	< 0.000
10–13	187 (56.8)	142 (43.2)		216 (65.7)	113 (34.3)	
14–17	74 (65.5)	39 (34.5)		82 (72.6)	31 (27.4)	
**Class**						
Class one	28 (28.3)	71 (71.7)	< 0.000	21 (21.2)	78 (78.8)	< 0.000
Class two	51 (45.9)	60 (54.1)		59 (53.2)	52 (46.8)	
Class three	51 (51.5)	48 (48.5)		64 (64.6)	35 (35.4)	
Class four	39 (47)	44 (53)		45 (54.2)	38 (45.8)	
Class five	66 (61.7)	41(38.3)		81 (75.7)	26 (24.3)	
Class six	51 (68.9)	23 (31.1)		58 (78.4)	16 (21.6)	
Class seven	55 (72.4)	21 (27.6)		54 (71.1)	22 (28.9)	
**Residency(years)**						
≤ 5	5 (38.5)	8 (61.5)	< 0.000	6 (46.2)	7 (53.8)	< 0.000
6–9	85 (39.4)	131 (60.6)		87 (40.3)	129 (59.7)	
10–13	179 (57.4)	133 (42.6)		207 (66.3)	105 (33.7)	
14–17	72 (66.7)	36 (33.3)		82 (75.9)	26 (24.1)	
**Schools**						
Longa Primary School	138 (60)	92 (40)	0.009	171 (74.3)	59 (25.7)	< 0.000
Nyengedi Primary School	141 (50.5)	138 (49.5)		153 (54.8)	126 (45.2)	
Nyangamara Primary School	62 (44.3)	78 (55.7)		58 (41.4)	82 (58.6)	

### Water, sanitation and hygiene factors associated with urogenital schistosomiasis among study participants

The majority of the participants (92.3%) reported that they had visited water bodies, and the river was the most visited water body by more than half (57.9%) of the participants. Of the participant who visited water bodies, 96% reported playing in the water, and more than half (57.9%) reported urinating while playing. Also, the majority (92.3%) reported doing activities in water bodies with washing clothes, washing dishes, and fetching water being the leading activities. The high prevalence of urogenital schistosomiasis was observed among children who visited (94.2%) and play (94.7%) in water bodies, urinate in water when swimming (59%), and don’t wear shoes (58.7%) when in or crossing water bodies (Table 7).

**Table 7:** Water, sanitation and hygiene status of the study participants (n = 649).

Variable	n (%)	*S*.*haematobium* positive	p-value
**Visit water body**			
Yes	599 (92.3)	322 (94.2)	0.061
No	50 (7.7)	20 (5.8)	
**Water body visited**			
Dam	104 (17.4)	48(14.9)	0.320
Pond water	95 (15.9)	49(15.2)	
Irrigation scheme	26 (4.3)	17(5.3)	
River	347 (57.9)	194 (60.2)	
Spring	27 (4.5)	14(4.3)	
**Source of water at school**			
Tap	466 (77.8)	253(78.6)	0.771
Deep well	41 (6.8)	22(6.8)	
Ponds	2 (0.3)	1(0.3)	
Shallow well	31 (5.2)	12(3.7)	
Tap and river	7 (1.2)	1(0.3)	
Tap and deep well	50 (8.3)	4(1.2)	
Do not know	2(0.3)	29(9)	
**Play in water**			
Yes	575 (96)	305(94.7)	0.087
No	24 (4)	17(5.3)	
**Urinate in water when playing**			
Yes	334 (57.9)	190(59)	0.030
No	243 (42.1)	115(35.7)	
**Wearing of shoes**			
Yes	247 (41.2)	133(41.3)	0.971
No	352 (58.8)	189(58.7)	
**Enough toilets at school**			
Yes	105 (16.2)	50(14.6)	0.255
No	544 (83.8)	292(85.4)	
**Place of urination when at school**			
At the School toilet	537 (82.7)	277(81)	0.161
In the bush	99 (15.3)	55(16.1)	
I run at home	13 (2)	10(2.9)	
**Doing activities in water bodies**			
Yes	599 (92.3)	321(93.9)	0.115
No	50 (7.7)	21(6.1)	
**Name of the activities**			
Agricultural activities	40 (6.7)	20(6.2)	0.328
Fishing	21 (3.5)	14(4.4)	
Washing clothes and dishes	130 (21.7)	64(19.9)	
Fetching water	150 (25)	82(25.5)	
Washing clothes, dishes and fetching water	211 (35.2)	110(34.3)	
All of the mentioned activities	47 (7.8)	31(9.7)	

### Factors associated with the ongoing transmission of urogenital schistosomiasis among the study participants

Table 8 summarizes the univariate and multivariate analysis of the factors associated with the ongoing transmission of urogenital schistosomiasis. The ongoing transmission of urogenital schistosomiasis was associated with the sex of the child, years of residency, location of the attended schools, practices towards urogenital schistosomiasis, and urinating in the water body. After adjusting for the confounders, the odds of transmitting the disease were high among children who resided at the Mtama district for 10 to13 years (AOR: 21.79, 95% CI: 1.37–346.4).

**Table 8:** Factors associated with ongoing transmission of urogenital schistosomiasis among the study participants (n = 649).

Variable	Univariate analysis		Multivariate analysis	
	^a^COR (95% CI)	p-value	^b^AOR (95% CI)	p-value
**Sex**				
Male (ref)	1.		1	
Female	0.7 (0.50–0.97)	0.032	0.704 (0.50–1.0)	0.042*
**Age (years)**				
6–9 (ref)	1		1	
10–13	0.08 (0.01–1.32)	0.078	0.09 (0.01–1.53)	0.097
14–17	0.41 (0.03–5.09)	0.485	0.41 (0.03–5.09)	0.486
**Class**				
Class one (ref)	1		1	
Class two	0.95 (0.37–2.44)	0.918	0.94 (0.11–7.9)	0.954
Class three	0.71 (0.31–1.66)	0.431	0.60 (0.24–1.45)	0.254
Class four	0.71 (0.33–1.54)	0.388	0.60 (0.27–1.31)	0.196
Class five	0.67 (0.31–1.42)	0.292	0.59 (0.27–1.30)	0.190
Class six	0.54 (0.27–1.08)	0.081	0.51 (0.24–1.03)	0.062
Class seven	0.78 (0.39–1.57)	0.480	0.75 (0.36–1.53)	0.423
**Residency(years)**				
≤ 5 (ref)	1		1	
6–9	2.3 (0.2–26.6)	0.506	2.4 (0.21–27.89)	0.480
10–13	23.44 (1.47–374.9)	0.026	21.79 (1.37–346.4)	0.029*
14–17	3.3 (0.25–43)	0.363	3.3 (0.26–43.1)	0.359
**Schools**				
Longa Primary School (ref)	1		1	
Nyengedi Primary School	0.29 (0.18–0.46)	<0.000	0.30 (0.17–0.53)	<0.000*
Nyangamara Primary School	0.34 (0.22–0.54)	<0.000	0.32 (0.20–0.52)	<0.000*
**Level of knowledge**				
Low level (ref)	1		1	
Moderate level	2.1 (0.82–5.13)	0.123	1.32 (0.47–3.67)	0.595
High level	1.7 (0.73–4.07)	0.215	1.41 (0.55–3.63)	0.474
**Classification of attitudes**				
Positive attitudes	1			
Negative attitudes	1.1 (0.79–1.63)	0.482		
**Classification of practices**				
Appropriate practices	1		1	
Inappropriate practices	0.6 (0.42–0.87)	0.006	0.88 (0.58–1.3)	0.039*
**Ever take praziquantel**				
Yes	0.7 (0.44–1)	0.060	1.7 (0.21–12.0)	0.626
No (ref)	1		1	
**Uptake of praziquantel in the last round**				
Yes	2.5 (0.26–24.1)	0.432		
No	3.8 (0.381–37.1)	0.256		
Don’t remember (ref)	1			
**Visit water bodies**				
Yes	0.6 (0.32–1.03)	0.064	0.264(0.05–1.3)	0.101
No (ref)	1		1	
**Type of water body visited**				
Dam (ref)	1			
Pond	1.256 (0.54–2.9)	0.598		
Irrigation scheme	1.0 (0.43–2.4)	0.980		
River	0.6 (0.19–1.7)	0.319		
Spring	0.9 (0.39–1.9)	0.683		
**Play in water bodies**				
Yes	0.9 (0.54–1.4)	0.627		
No (Ref)	1			
**Urinating in water body**				
Yes	0.7(0.5–1)	0.028	2.1 (0.71–6.3)	0.018*
No (Ref)	1		1	
**Wearing of shoes**				
Yes	1.0 (0.7–1.4)	0.971		
No (Ref)	1			
**Doing activities in water bodies**				
Yes	0.6 (0.35–1.1)	0.117	1.0 (0.24–4.3)	0.985
No (Ref)	1		1	
**Name of the activities**				
Agricultural activities (ref)	1		1	
Fishing	1.9 (0.82–4.6)	0.134	1.6 (0.63–4.2)	0.306
Washing clothes and dishes	1 (0.33–2.9)	0.954	0.9 (0.28–2.9)	0.859
Fetching water	2 (1–4)	0.051	1.3 (0.59–2.9)	0.514
Washing clothes, dishes and fetching water	1.6 (0.8–3.2)	0.174	1 (0.47–2.3)	0.918
All of the mentioned activities	1.8 (0.92–3.5)	0.088	0.9 (0.41–1.9)	0.734

^*a*^*COR* Stands for Crude Odds Ratios

^*b*^*AOR* Stands for Adjusted Odds Ratios

*Statistical significance at p<0.05.

### Malacological survey

A total of 947 fresh water snails were collected from 15 sampling sites in the selected three villages. Of the 947 snails collected, 627 (66.2%) were *Bulinus globosus*, and 320 (33.8%) were *Bulinus nasutus* based on the shell morphology. The highest number of snails were collected at Mtua longa village (54%), followed by Nyangamara village (27.5%) and Nyengedi village (18.5%). A total of 18 (1.9%) snails were infected. Mtua longa village (0.84%) had a high number of *Bulinus* spp who shed cercariae, followed by Nyengedi village (0.74%) and Nyangamara village (0.32%). The natural prevalence of shedding schistosome cercariae was 2.2% in *B*.*globosus* and 1.3% in *B*.*nasutus* (Table 9).

**Table 9:** Summary of the distribution of snails and prevalence of cercariae shedding per site (n = 947).

Village	Name and type of the site	Snail species collected		Total number of snails collected	Number of snails infected (%)	
		*B*.*globosus*	*B*.*nasutus*		*B*.*globosus*	*B*.*nasutus*
Mtua longa	Mtua longa spring	-	-	0	-	**-**
	Kitumba stream point A	139	-	139	-	**-**
	Kitumba stream point B	53	-	53	-	**-**
	Mnongo stream point A	-	-	0	-	**-**
	Mnongo stream point B	93	53	146	4(4.3)	2(3.8)
	Mnongo stream point C	54	27	81	2(3.7)	**-**
	Longa river	46	47	93	**-**	**-**
Nyengedi	Nyengedi River point A	-	**-**	0	**-**	**-**
	Nyengedi River point B	55	**-**	55	**-**	**-**
	Nyengedi River point C	43	**-**	43	5 (11.6)	**-**
	Rice paddy field	32	45	77	1(3.1)	1(2.2)
Nyangamara	Mbawe Dam	21	**-**	21	-	-
	Rice paddy field A	26	61	87	-	-
	Rice paddy field B	34	69	103	-	-
	Rice paddy field C	31	18	49	2(6.5)	1(5.5)
**Total**		**627**	**320**	**947**	**2.2**	**1.3**

## Discussion

In this present study, the overall prevalence and intensity of urogenital schistosomiasis among school-aged children in Mtama Lindi was 52.7% and 90.23±241.67 EPG, respectively, which indicates a high-risk community based on the WHO categories of endemic communities [22]. Despite twelve rounds of praziquantel distribution in the Mtama district, the prevalence of the disease has remained high, nearly similar to the prevalence of 58.9% reported in 1987 [9]. The high prevalence could be contributed by the absence of praziquantel distribution in the last year due to interruption of mass drug administration activities because of the coronavirus disease-2019. In addition, haematuria, both non-visible (microhaematuria) and visible (macrohaematuria), was identified, and the prevalence was 13.1% and 46.2%, respectively. The observed haematuria is a morbidity indicator that calls for the urgent need for treatment of the infected children because if they are not treated, the intensity will increase and might result in chronic sequelae.

The prevalence of urogenital schistosomiasis in this study increased as the age groups of the children increased attributed to early exposure to infested water which results in the acquisition of the disease at an early age with peak prevalence and intensity concentrated in the 6 to 17 years. The present findings are consistent with the other studies reported in Tanzania and Nigeria [23,24]. Also, there was a statistically significant variation of urogenital schistosomiasis prevalence among the schools with the highest prevalence in Longa Primary School (60.9%) followed by Nyengedi (55.9%) and Nyangamara (32.9%) Primary Schools. This could be attributed to the abundance of *Bulinus* spp snails and the proximity of the school/village to transmission sites as Mtua longa village was closest to the water bodies, followed by Nyengedi, and lastly Nyangamara.

In this study, it was noticed that not all children with symptoms of urogenital schistosomiasis were investigated before treatment in the health facilities instead, their parents bought drugs at the local pharmacies. The reasons for doing this were to reduce the costs for doctors’ consultation, laboratory investigation, and treatment. However, the praziquantel was not sold in pharmacies thus; the parents, usually bought antibiotics (Metronidazole and Ciprofloxacin) instead of praziquantel. The finding is consistent with similar studies conducted in Zanzibar, Kenya, and Nigeria [25–28]. This practice is not good because children are not treated for schistosomiasis and are at high risk of developing morbidity associated with the disease and antimicrobial resistance.

There is high self-reported uptake of praziquantel in this community, above 78.9%, presumably due to more than a decade of praziquantel mass drug administration. The ongoing mass drug administration of praziquantel for more than a decade in Mtama could potentially lead to the reduced efficacy of the drugs, which portends the selection of the drug-resistant. There was a group of 104 students who reported that they had never swallowed praziquantel, of which 99 children were standard one. The standard one children in the last round of praziquantel distribution were not qualified for praziquantel uptake because they were preschoolers. In this group of the 99 children who never took praziquantel, 43 children were urogenital schistosomiasis positive. There is evidence that standard one students probably get infected through early exposure to infested water bodies when under five years, and contribute to the reservoir of continuity of transmission in this community [29,30].

All children have heard of urogenital schistosomiasis; this is because the disease was highly endemic in the district. Knowledge on the cause of urogenital schistosomiasis, risk factors, and mode of transmission can generate effective change in communities by reducing risk behavior practices and increase their participation in the control interventions [31]. However, the findings of this study revealed a group of students with a low level of knowledge (31.1%), negative attitudes (47.5%), and inappropriate practices (41.1%) on urogenital schistosomiasis. There is a high chance for this group to compromise the ongoing effort to control urogenital schistosomiasis with an increased risk of the disease’s transmission in Mtama district. In sub-Saharan Africa, for example; lack of knowledge, negative attitude, and risky water practices were associated with the persistence of urogenital schistosomiasis transmission [32].

Inadequate water supply, poor sanitation, and unhygienic practices are among the risk factors for the transmission of urogenital schistosomiasis in endemic settings [4,33]. Mtama district has a scarcity of clean and safe water, which causes some community members to depend on other sources of water such as irrigation schemes, rivers, dams, and shallow wells [15]. Therefore, when children are at home, they have a tendency to visit and assist chores in water sources and playing in the water, thus, exposed to the infested water and the reason for the observed high prevalence of the disease in this group. In addition, the community has households without an improved form of toilets [14]. Hence, stimulating unhygienic practices perpetuating the transmission of the diseases that’s why children with unhygienic practices such as open urination in water sources and the habit of walking barefooted were highly infected compared to the rest. The findings are comparable with the previous studies done in Tanzania, Sudan, Nigeria, Senegal [34–37].

The factors which were predictive of infection in this study were sex of the child, years of residency, school location, practices towards urogenital schistosomiasis, and urinating in the water body. This observation conforms to several studies reported in Sub-Saharan Africa [34,35,38–40]. Of the factors mentioned above, only years of residency remained a significant predictor of urogenital schistosomiasis transmission in the multivariate logistic regression analysis. The higher odds of infection were observed among the children who resided in the Mtama community for 10 to 13 years. The years of residency corresponded with the years of the children because most of the children were born and raised in the Mtama district. The children of 10 to 13 years are probably the most active, and being the residents of the respective villages for that long, they seem to know and visit most of the surrounded water bodies frequently. Hence, the high risk of transmitting and acquiring urogenital schistosomiasis.

In a malacological survey, the availability of *B*.*globosus* and *B*.*nasutus* snails in the Mtama district is the important epidemiological factor for urogenital schistosomiasis transmission. Also, the recovery of the infective stage (cercariae) in *B*.*globosus* (2.2%) and *B*.*nasutus* (1.3%) is a sign that there is ongoing transmission of the urogenital schistosomiasis in this community. From this study, the identified hotspots for urogenital schistosomiasis transmission were the Mnongo stream for the Longa community, the Nyengedi river, and rice paddy fields for the Nyengedi community, and rice paddy fields for the Nyangamara community. The prevalence of cercariae shedding overlaps with the prevalence of the disease in this community. Hence, the highest prevalence of the disease was observed in the community, with the highest number of *Bulinus spp* snails that shed cercariae. The findings of this study are in line with the studies conducted in Tanzania, Kenya, and Nigeria, which showed in urogenital schistosomiasis highly endemic areas also the prevalence of cercariae shedding are high [23,41,42]. This calls for the urgent need to map all the transmission sites in the district and the initiation of the snail control intervention. The observed low prevalence (1.9%) of the infected *Bulinus* snails was responsible for a high prevalence (52.7%) of *S*. *haematobium* among school-aged children in the Mtama District. This observation is similar to the findings of the study conducted in Tanzania [41]. This could be as a result of collecting the snails only once and the use of cercariae shedding methods which could miss many prepatent infections. If the molecular techniques were used for snails screening, it was more likely to detect a considerably higher number of the infected *Bulinus* snails [43,44].

This study had the following limitations; the recall bias, the questionnaire consisted of questions on the history of urogenital schistosomiasis sickness and treatment among the study participants, such as the history of ever and when suffered the disease and praziquantel uptake in the previous years; this might results in under-or-over reporting hence, affected the accuracy of the collected information. Another limitation, the snails were collected only once, which might have underestimated the prevalence of cercariae shedding among the infected *Bulinus spp* snails. In addition, only a single urine sample was collected in each participant that might underestimate the prevalence and intensity of urogenital schistosomiasis. There is a possibility that the actual prevalence is higher than what was observed in this study.

## Conclusions and recommendations

This study revealed a high prevalence of urogenital schistosomiasis among school-aged children in Mtama in Lindi despite more than 12 rounds of praziquantel distribution. The factors perpetuating the ongoing transmission of the urogenital schistosomiasis in the study area include; the absence of praziquantel distribution for the past one year, inadequate knowledge, coupled with negative attitudes and inappropriate practices on the disease transmission, treatment, and prevention, inadequate supply of piped water, poor sanitation facilities, unhygienic practices, intense human water contact activities especially in rivers and presence of infected *Bulinus spp* snails. Therefore, there is a need for the bi-annual distribution of praziquantel in all populations at risk to speed up the control of the disease in the Mtama district. In addition, it’s necessary for the neglected tropical diseases control programme to initiate snail control intervention and integrate with the health education for behavioral change. Also, the government should ensure that there is an adequate supply of clean water and sanitation facilities in the community. For sustainable control of urogenital schistosomiasis, the recommended measures should be integrated and implemented together. Lastly, further studies are required to establish why only long residency is a significant risk factor for the continuity of transmission.

## Supporting information

S1 File Questionnaire Kiswahili. (DOCX)

S2 File Questionnaire English. (DOCX)

S1 Data The data set used for analysis. (XLSX)
