# Supplementary material for: Persistence of Schistosoma haematobium transmission among school children and its implication for the control of urogenital schistosomiasis in Lindi, Tanzania
Source: PLoS One. 2022 Feb 15;17(2):e0263929. doi: 10.1371/journal.pone.0263929 (PMC8846507; doi:10.1371/journal.pone.0263929)
Supplement: S1 File — (DOCX) [file pone.0263929.s001.docx]

# S1 File-Kiswahili questionnaire

Namba ya utambulisho ya hojaji …………………………

Jina la mhojaji ………………………..

Tarehe ya mahojiano ……………………………..

**SEHEMU A: Taarifa za Kidemografia**

| 1 | Jina la Kata |  |
| --- | --- | --- |
| 2 | Jina la kijiji |  |
| 3 | Jina la Shule |  |
| 3 | Jinsia | Mwanaume…………1  Mwanamke ...……….2 |
| 4 | Umri (miaka) |  |
| 6 | Darasa analosoma |  |
| 8 | Muda ulioishi katika kijiji hiki |  |

**SEHEMU B**

**Ufahamu juu ya ugonjwa wa kichocho cha mkojo**

1. Je unajua ugonjwa unaoitwa kichocho cha mkojo?
2. Ndiyo…………………………………….1
3. Hapana…………………………………..2
4. Kama ndio unaufahamu, je ulipata wapi taarifa kuhusiana na ugonjwa huu?
5. Nyumbani…... ……………….1
6. Vyombo vya habari…..…..…...2
7. Shuleni…………………….….3
8. Zahanati…………………….…4
9. Rafiki……………….….……...5
10. Kwingine (taja)…………..........6
11. Je ugonjwa unaambukizwa kwa njia ipi?
12. Kunywa maji machafu …………………………………………………….…..1
13. Kuogelea na kucheza kwenye maji yenye vimelea vya ugonjwa……………...2
14. Kupeana mikono ………………………………………………………………3
15. Kula chakula kichafu……………………………………………………….….4
16. Kucheza na udongo……………………………………………………………5
17. Sijui…………………………………………………………………………….6
18. Je, konokono wanahusika katika kusambaza vimelea vya kichocho cha mkojo?
19. Ndiyo.………………..1
20. Hapana……..………...2
21. Sijui…………………..3
22. Je zipi ni dalili za ugonjwa wa kichocho cha mkojo?
23. Kikohozi ……………………………………….1
24. Kuwashwa ……………………………………..2
25. Kuumwa kichwa ………………..……………..3
26. Homa…………………………………………...4
27. Maumivu ya tumbo ……….……………………5
28. Damu kwenye mkojo…………………………...6
29. Damu kwenye choo …………………………....7
30. Kuharisha……………………………………….8
31. Sijui…………………………………………..…9
32. Je mtu mwenye kichocho cha mkojo anaweza kutibiwa na kupona?
33. Ndiyo………………….…1
34. Hapana………..………….2
35. Sijui …………………...…3

1. Ni kwa njia gani kichocho cha mkojo kinaweza kutibiwa?
2. Kwa kumeza vidonge ……………….1
3. Kuchomwa sindano..………………...2
4. Kufanyiwa upasuaji …………………3
5. Kwa kutumia dawa asilia…………….4
6. Sijui…………………………………..5
7. Je kichocho cha mkojo kinazuilika?
8. Ndiyo….………………1
9. Hapana………………...2
10. Sijui……………………3
11. Ni kwa jinsi gani unaweza kuzuia na kudhibiti ugonjwa wa kichocho cha mkojo?
12. Kwa kutumia dawa ya kutibu kichocho……………………...…………....1
13. Kuepuka kugusana/kutumia vyanzo vya maji visivyo salama ….…………2
14. Kutumia maji ya bomba ……………………………………………………3
15. Kutumia vyoo ………………………………………………………………4
16. Kuimarisha usafi binafsi …………………………………………….……...5
17. Sijui………………………………………………………………………….6

**SEHEMU C: Historia ya matibabu ya kichocho (Kama hajawahi kuumwa ruka swali la 9 mpaka swali la 13)**

1. Je umewahi kuugua ugonjwa wa kichocho cha mkojo?
2. Ndiyo………………………………...1
3. Hapana……………………………….2
4. Sikumbuki……….. ………………….3
5. Ni lini uliugua?
6. Naumwa sasa hivi ……………………………………………………..1
7. Mwezi mmoja uliopita …….…………………………………………..2
8. Zaidi ya miezi mitatu uliyopita lakini chini ya miezi sita……………...3
9. Zaidi ya miezi sita iliyopita ……….………………...............................4
10. Mwaka mmoja uliopita ……………………….………………………..5
11. Sikumbuki………………………………………………………………6
12. Je ulienda ukapatiwa huduma ya uchunguzi katika kituo cha afya?
13. Ndiyo…………………….…1
14. Hapana………………..…….2
15. Sikumbuki ………………….3
16. Je ulipatiwa matibabu?
17. Ndiyo………………….……………1
18. Hapana…………………..………….2
19. Sikumbuki………………….……….3
20. Je wazazi wako huwa wanakuruhusu kumeza dawa ya kuzuia kichocho pale zinapoletwa shuleni?
21. Ndiyo…………………………………1
22. Hapana………………………………..2

14 Kama hapana zipi ni sababu za kunyimwa kumeza dawa……………………

1. Je umeshawahi kumeza dawa za kuzuia kichocho zinapogawiwa shuleni?
2. Ndiyo…………………………………1
3. Hapana………………………………..2
4. Je ulimeza dawa za kuzuia kichocho cha mkojo zilivyo gawiwa mara ya mwisho?
5. Ndiyo………………….……………1
6. Hapana…………………..………….2
7. Sikumbuki………………….……….3
8. Kama hapana ipi ilikuwa sababu ya kutokumeza dawa…………………………

**SEHEMU D: Mahusiano kati ya maji, usafi wa mazingira na usafi binafsi unavyohusiana na maambukizi ya kichocho cha mkojo**

1. Je huwa unatembelea chanzo chochote cha maji kilichopo karibu na shule au nyumbani?
2. Ndiyo…………………………………………..1
3. Hapana………………………………………...2
4. Kama jibu lako ni ndiyo, je ni vyanzo vipi vya maji unavyotembelea mara kwa mara?
5. Bwawa ………………………………………….1
6. Madimbwi………….……………………….…..2
7. Mfumo wa umwagiliaji……………………...…..3
8. Mto………….…………………………………..4
9. Chemchem.......………………………………….5
10. Nyingine (taja)……………..……………………6
11. Ni chanzo kipi cha maji mnatumia shuleni?
12. Bomba ……………………….1
13. Kisima ……………………….2
14. Madimbwi …………………...3
15. Visima vifupi ………………...4
16. Mto…………………………...5
17. Sijui……………………...........6
18. Je huwa mnacheza kwenye vyanzo vya maji vilivyopo kijijini/shuleni hapa?
19. Ndiyo………………………1
20. Hapana..…..………………..2
21. Je uwa mnakojoa kwenye maji wakati mnaogelea?
22. Ndiyo…………………………………1
23. Hapana………………………………..2
24. Je huwa unavaa viatu mnapotembea kwenye kingo za maji?
25. Ndiyo………………………………...1
26. Hapana……………………………….2
27. Je mna vyoo vya kutosha shuleni?
28. Ndiyo………………………………...1
29. Hapana……………………………….2
30. Je mnaendaga wapi kukojoa wakati wa mapumziko au wakati wowote unapojisikia kukojoa?
31. Kwenye vyoo vilivyopo shuleni…………………….1
32. Kwenye kichaka …………………………………….2
33. Nakimbia nyumbani…………………………………3
34. Naenda karibia na chanzo cha maji …………………4
35. Kwingine (taja)………………………………………5
36. Je nyumbani huwa unasaidia kazi yoyote inayokupelekea kwenda kwenye vyanzo vya maji?
37. Ndiyo…………………………………...1
38. Hapana………………………………….2
39. Je ni kazi zipi hizo?
40. Kazi za kilimo……………………………….….1
41. Uvuvi ……………………………………….….2
42. Kufua nguo na kuosha viombo…………………3
43. Kuchota maji……………………………………4
44. Nyingine (taja)…………………………….....…5

**SEHEMU E: Maswali ya mwelekeo [Nitasoma sentensi kadhaa zinazohusiana na ugonjwa wa kichocho cha mkojo, tafadhali chagua kiwango unachokubaliana au kukataa kwa kila sentensi.]**

| **NAMBA** | **SWALI** | **Sikubaliani kabisa** | **Sikubaliani** | **Sinauwakika** | **Nakubaliana** | **Nakubaliana**  **Kabisa** |
| --- | --- | --- | --- | --- | --- | --- |
| 1 | Kichocho cha mkojo ni ugonjwa mbaya. | **1** | **2** | **3** | **4** | **5** |
| 2 | Kichocho cha mkojo ni ugonjwa unaotibika. | **1** | **2** | **3** | **4** | **5** |
| 3 | Kichocho cha mkojo kinazuilika. | **1** | **2** | **3** | **4** | **5** |
| 4 | Haijalishi nikikojoa kwenye maji. | **1** | **2** | **3** | **4** | **5** |
| 5 | Watoto wadogo hawawezi kupata kichocho cha mkojo. | **1** | **2** | **3** | **4** | **5** |
| 6 | Kukojoa damu ni sehemu ya ukuaji. | **1** | **2** | **3** | **4** | **5** |
| 7 | Kuna mahusiano kati ya kukojoa damu na imani za kishirikina. | **1** | **2** | **3** | **4** | **5** |
| 8 | Ni muhimu kupima kichocho cha mkojo mara kwa mara. | **1** | **2** | **3** | **4** | **5** |
| 9 | Ni muhimu kumeza vidonge vya kuzuia maambukizi ya kichocho cha mkojo. | **1** | **2** | **3** | **4** | **5** |
| 10 | Maambukizi ya ugonjwa wa kichocho cha mkojo yanaweza kujitokeza tena mda mfupi baada ya matibabu. | **1** | **2** | **3** | **4** | **5** |

**SEHEMU F: Maswali ya mazoea/tabia [Nitasoma sentensi kadhaa zinazohusiana na ugonjwa wa kichocho cha mkojo, tafadhali chagua kiwango unachokubaliana au kukataa kwa kila sentensi.]**

| **NAMBA** | **SWALI** | **Sikubaliani kabisa** | **Sikubaliani** | **Sina uwakika** | **Nakubaliama** | **Nakubaliana kabisa** |
| --- | --- | --- | --- | --- | --- | --- |
| 1 | Mtu anaweza kupata kichocho kwa kutumia maji yenye vimelea vya ugonjwa kwenye mito/ mabwawa kwa shughuli mbalimbali za nyumbani. | **1** | **2** | **3** | **4** | **5** |
| 2 | Mtoto anaweza kupata kichocho cha mkojo kwa kuogelea au kucheza kwenye maji ya mto/bwawa | **1** | **2** | **3** | **4** | **5** |
| 3 | Mtoto anaweza kupata kichocho kwa kufuvuka mto miguu peku bila kuvaa viatu. | **1** | **2** | **3** | **4** | **5** |
| 4 | Kukojoa kwenye vyanzo vya maji kunaweza kusababisha maambukizi kutokea. | **1** | **2** | **3** | **4** | **5** |
| 5 | Mtu anaweza kupata kichocho cha mkojo anapokuwa bwawani/mtoni anafua nguo au kuosha vyombo. | **1** | **2** | **3** | **4** | **5** |
| 6 | Mtu anaweza kupata ugonjwa kwa kunywa maji yasio safi na salama. | **1** | **2** | **3** | **4** | **5** |
| 7 | Mtu anaweza kuzuia maambukizi ya ugonjwa kwa kuchemsha maji kwa ajili ya kumuogesha mtoto. | **1** | **2** | **3** | **4** | **5** |
| 8 | Maambukizi ya ugonjwa wa kichocho cha mkojo yanaweza kuzuilika kwa kuua konokono | **1** | **2** | **3** | **4** | **5** |
| 9 | Tiba asilia ni matibu yenye ufanisi zaidi kwenye kutibu kichocho cha mkojo. | **1** | **2** | **3** | **4** | **5** |
| 10 | Matumizi ya nguo zinazozuia maji kupenya, mtu anapokuwa kwenye vyanzo vya maji yanaweza kuzuia mtu asipate maambukizi. | **1** | **2** | **3** | **4** | **5** |
