# Supplementary material for: Persistence of Schistosoma haematobium transmission among school children and its implication for the control of urogenital schistosomiasis in Lindi, Tanzania
Source: PLoS One. 2022 Feb 15;17(2):e0263929. doi: 10.1371/journal.pone.0263929 (PMC8846507; doi:10.1371/journal.pone.0263929)
Supplement: S2 File — (DOCX) [file pone.0263929.s002.docx]

# S2 File-English questionnaire

Questionnaire ID no…………………………

Name of Interviewer………………………..

Date of Interview……………………………..

**PART A**

**Demographic information**

| 1 | Ward Name |  |
| --- | --- | --- |
| 2 | Village Name |  |
| 3 | Name of school |  |
| 3 | Sex | Male…………………1  Female……………….2 |
| 4 | Age (years) |  |
| 6 | Class |  |
| 8 | Duration lived in the village |  |

**PART B**

**Knowledge of Urogenital Schistosomiasis**

1. Do you know the disease called urinary schistosomiasis?
2. Yes…………………………………….1
3. No……………………………………..2
4. If yes, where did you get the information about urinary schistosomiasis?
5. Home…... ……………………………….1
6. Mass media……………………………...2
7. School…………………………………...3
8. Dispensary………………………………4
9. Friend…………………………………...5
10. Others (specify)………………………....6
11. How is the disease transmitted?
12. By drinking dirty water…………………………………….1
13. By swimming and playing in infested water ……………....2
14. By shaking hands …………………………………………..3
15. By eating contaminated food……………………………….4
16. By playing with soil…………………………………………5
17. Do not know……………………………………………........6
18. Others(specify)………………………………………………7
19. Snails play part in the transmission of urogenital schistosomiasis
20. Yes………………………..1
21. No………………………...2
22. Do not know………………3
23. What are the signs/symptoms of the disease?
24. Coughing ……………………………………….1
25. Itching ………………………………………….2
26. Headache ……………………………………….3
27. Fever ……………………………………………4
28. Stomachache ……………………………………5
29. Urine in blood …………………………………..6
30. Blood in feces …………………………………..7
31. Diarrhea…………………………………………8
32. Do not know…………………………………….9
33. Can someone with urinary schistosomiasis be cured?
34. Yes ……………..…………………………….1
35. No ……………………………………………2
36. Do not know….………………………………3
37. How can it be treated?
38. By swallowing tablets ………………………………..…...1
39. By injection ………………………………..……………...2
40. By being operated ………………………………………...3
41. By traditional medicine……………………………………4
42. Do not know……………………………………………….5
43. Is urogenital schistosomiasis a preventable disease?
44. Yes……………………………...1
45. No…………………………….2
46. Do not know…………………….3
47. How can you prevent and control schistosomiasis?
48. Treatment using anti-schistosomal medicine……………………...………..1
49. By avoiding contact with unprotected water bodies…………………….….2
50. Use of pipe water……………………………………………………………3
51. Use of latrines ………………………………………………………………4
52. By improving personal hygiene….……………………..…..……..…..….....5
53. Do not know…………………………………………………………………6

**PART C**

**Treatment history (If a student has never acquired the disease skip question 9 to 13)**

1. Have you have ever suffered urinary schistosomiasis?
2. Yes…………………………………1
3. No………………………………….2
4. Do not remember ………………….3
5. When did you suffer?
6. Currently, I am suffering ….……………………………………...1
7. A month ago ……………………………………………………..2
8. More than 3 months ago but less than six months ………………3
9. More than six months ago ………………………………….........4
10. A year ago ……………………………………………………….5
11. I do not remember………………………………………………..6
12. Did you get investigated in the health facility?
13. Yes…………………………………1
14. No………………………………….2
15. Do not remember ………………….3
16. Did you get treatment?
17. Yes…………………………………1
18. No………………………………….2
19. Do not remember ………………….3
20. Did your parents allow you to take praziquantel in the school campaigns?
21. Yes…………………………………1
22. No………………………………….2
23. If no what were the reasons………………………………………….
24. Have you ever taken praziquantel when distributed at school?
25. Yes…………………………………1
26. No………………………………….2
27. Did you take praziquantel in the last round of distribution?
28. Yes…………………………………1
29. No………………………………….2
30. Do not remember…………………...3
31. If no what was the reason………………………………………….

**PART D**

**WASH practices associated with transmission of urogenital schistosomiasis**

1. Do you visit any water bodies around the school or home?
2. Yes…………………………………1
3. No………………………………….2
4. If yes which water body do you come into contact with frequently?
5. Dam………………………….…...1
6. Pond water……………………….2
7. Irrigation scheme ……………...…3
8. River………………………………4
9. Spring……………………………..5
10. Others (specify)…………….……..6
11. Which water source do you depend on at school?
12. Tap water ……………………….1
13. Deep well ……………………….2
14. Ponds …………………………...3
15. Shallow well ……………………4
16. River ……………………………5
17. Do not know…………………….6
18. Do you play and swim in the surface water present nearby the village/school?
19. Yes…………………………………1
20. No………………………………….2
21. Sometimes………………………….3
22. Do you urinate in the water while swimming?
23. Yes…………………………………1
24. No………………………………….2
25. Do you wear shoes while walking along the shores of the dam?
26. Yes…………………………………1
27. No………………………………….2
28. Do you have enough toilets at school?
29. Yes…………………………………1
30. No………………………………….2
31. Where do you go for urination during break time or whenever you feel to urinate?
32. At the School toilet……………………1
33. In the bush …………………………….2
34. I run at home…………………………...3
35. At the nearby water source…………….4
36. Others (specify)………………………...5
37. Do you participate in any activities at home that expose you to contact with water?
38. Yes…………………………………1
39. No………………………………….2
40. What are those activities?
41. Agricultural activities……………………………..1
42. Fishing ……………………………………………2
43. Washing clothes and dishes……………………….3
44. Fetching water ……………………………………4
45. Others (specify)………………………………..….5

**PART E**

**Attitudes on urogenital schistosomiasis [Am going to read several statements regarding urogenital schistosomiasis please rate the degree to which you agree or disagree with each]**

| **CODE** | **QUESTION** | **Strongly**  **Disagree** | **Disagree** | **Not sure** | **Agree** | **Strongly**  **Agree** |
| --- | --- | --- | --- | --- | --- | --- |
| 1 | Urogenital schistosomiasis is a serious disease. | **1** | **2** | **3** | **4** | **5** |
| 2 | Urogenital schistosomiasis is a curable disease. | **1** | **2** | **3** | **4** | **5** |
| 3 | Urogenital schistosomiasis is a preventable disease. | **1** | **2** | **3** | **4** | **5** |
| 4 | It doesn't matter if I urinate in the water. | **1** | **2** | **3** | **4** | **5** |
| 5 | Young children cannot acquire urogenital schistosomiasis. | **1** | **2** | **3** | **4** | **5** |
| 6 | Hematuria is a part of growing up. | **1** | **2** | **3** | **4** | **5** |
| 7 | There is an association between hematuria and witchcraft. | **1** | **2** | **3** | **4** | **5** |
| 8 | It's important to periodically screen for schistosomiasis. | **1** | **2** | **3** | **4** | **5** |
| 9 | It's important to take anti-schistosomiasis tablets when distributed in school | **1** | **2** | **3** | **4** | **5** |
| 10 | Urogenital schistosomiasis can reoccur soon after treatment. | **1** | **2** | **3** | **4** | **5** |

**PART F**

**Practices towards urogenital schistosomiasis [Am going to read several statements regarding urogenital schistosomiasis please rate the degree to which you agree or disagree with each]**

| **CODE** | **QUESTION** | **Strongly**  **Disagree** | **Disagree** | **Not sure** | **Agree** | **Strongly**  **Agree** |
| --- | --- | --- | --- | --- | --- | --- |
| 1 | One can acquire urogenital schistosomiasis by using infested water from a river/dam for domestic purposes. | **1** | **2** | **3** | **4** | **5** |
| 2 | Children can acquire infection by swimming/playing in a river/dam. | **1** | **2** | **3** | **4** | **5** |
| 3 | Children can acquire infection by crossing a river barefooted. | **1** | **2** | **3** | **4** | **5** |
| 4 | Urinating in water sources can cause the transmission to occur. | **1** | **2** | **3** | **4** | **5** |
| 5 | One can acquire the infection when washing clothes or utensils in open water sources. | **1** | **2** | **3** | **4** | **5** |
| 6 | One can be infected by drinking untreated water. | **1** | **2** | **3** | **4** | **5** |
| 7 | One can prevent infection by boiling water for bathing. | **1** | **2** | **3** | **4** | **5** |
| 8 | The transmission of urogenital schistosomiasis can be prevented by killing the snails | **1** | **2** | **3** | **4** | **5** |
| 9 | Traditional treatment is an effective way to treat urogenital schistosomiasis. | **1** | **2** | **3** | **4** | **5** |
| 10 | Use of protective waterproof clothes when in contact with water can prevent from acquiring the disease. | **1** | **2** | **3** | **4** | **5** |
